# Dynamic contrast-enhanced MRI and Apparent diffusion coefficient mapping in the characterization of Palpable breast lesions: A prospective observational study

**DOI:** 10.1186/s43055-023-01002-3

**Published:** 2023-05-22

**Authors:** Deb K. Boruah, Nitashree Konwar, Bidyut B. Gogoi, Karuna Hazarika, Halimuddin Ahmed

**Affiliations:** 1 Department of Diagnostic and Interventional Radiology, All India Institute of Medical Sciences, Guwahati, Assam 781101 India; 2 Department of Radio-Diagnosis, Tezpur Medical College and Hospital, Tezpur, Assam 784010 India; 3 Department of Pathology, Assam Medical College and Hospital, Barbari, Assam 786002 India

**Keywords:** DWI (diffusion-weighted imaging), DCE-MRI (dynamic contrast-enhanced magnetic resonance imaging), Kinetic curve, Breast carcinoma, BI-RADS

## Abstract

**Background:**

Breast MRI is the imaging modality of choice in patients with palpable breast lesions unequivocal on mammography and ultrasonography. This study aims to evaluate the role of dynamic contrast-enhanced MRI (DCE-MRI) and apparent diffusion coefficient mapping in the characterization and differentiation of various palpable breast lesions. This prospective observational study was conducted in a tertiary care hospital between July 2019 and June 2021. Sixty-six patients with palpable breast lesions were undergone MRI scans of the breasts. The various palpable breast lesions were categorized according to the 5th edition BI-RADS lexicon. The sensitivity of ADC mapping and DCE-MRI was determined for differentiation of various palpable breast lesions according to the BI-RADS category and gold standard histopathological findings.

**Results:**

Of 66 patients with palpable breast lesions, 36 patients (54.5%) were benign and 30 patients (45.5%) were malignant lesions. Malignant palpable breast lesions had a mean ADC value of 0.939 ± 0.166[SD] × 10^−3^ mm^2^/s, and benign lesions had 1.891 ± 0.524[SD] × 10^−3^ mm^2^/s where unpaired Student *t*-test showed statistically significant difference of *P* value 0.0005. BI-RADS 2 lesions had a mean ADC value of 2.056 ± 0.471[SD] × 10^−3^ mm^2^/s, BI-RADS 3 had 1.314 ± 0.151[SD] × 10^−3^ mm^2^/s, BI-RADS 4 had 0.935 ± 0.119[SD] × 10^−3^ mm^2^/s, and BI-RADS 5 had 0.930 ± 0.943[SD] × 10^−3^ mm^2^/s. BI-RADS 2 category showed optimal cutoff mean ADC of 1.508 × 10^−3^ mm^2^/s with a sensitivity of 85.7% and specificity of 94.7%, BI-RADS 3 lesions had 1.208 × 10^−3^ mm^2^/s with a sensitivity of 75% and specificity of 55.2%, BI-RADS 4 lesions had 1.064 × 10^−3^ mm^2^/s with a sensitivity 80% and specificity of 67.9%, and BI-RADS 5 lesions had 1.013 × 10^−3^ mm^2^/s with a sensitivity of 80% and specificity of 82.6%.

**Conclusions:**

Breast MRI is superior to the other imaging modalities for the characterization and differentiation of various palpable breast lesions. The combined use of ADC mapping and DCE-MRI had more sensitivity than conventional MRI, ADC mapping or DCE-MRI alone.

## Background

Breast cancer is the leading cause of morbidity and mortality in females [1]. Early detection of breast cancer with various imaging modalities like mammography, tomosynthesis, ultrasonography and MRI helps in the improvement of the patient’s prognosis [1]. MRI is the supplementary imaging modality of choice for suspicious or indeterminate breast lesions detected on mammography or ultrasound. Early detection of breast cancer with various imaging modalities and early breast biopsies helps in more institutions of conservative breast surgery [2–4].

The addition of newer MRI sequences like ADC mapping and DCE-MRI with conventional breast MRI sequences had important added advantages in the identification, characterization and differentiation of malignant and benign palpable breast lesions [5]. The use of DWI and apparent diffusion coefficient (ADC) value quantification helps in the detection and differentiation of benign and malignant breast lesions without injecting IV contrast injection in patients with renal dysfunction or prior contrast reaction [6]. DCE-MRI had more sensitivity with relatively low specificity for the differentiation of benign and malignant breast lesions [7]. However, the addition of DWI sequence and ADC mapping increases the specificity of DCE-MRI [8]. MRI had a sensitivity of 94–100% for the detection of invasive breast cancer [9]. MRI provides accurate visualization of retromammary space, axillae, chest wall and involvement of rib or sternum in the assessment of infiltrating breast cancers better than conventional breast imaging like USG and mammography, which implicates the patient management and subsequent prognosis [2, 6].

This study aims to evaluate the role of dynamic contrast-enhanced MRI (DCE-MRI) and apparent coefficient mapping in the characterization and differentiation of various palpable breast lesions.

## Methods

This prospective, observational study was conducted in a tertiary care hospital with 66 female patients with palpable breast lesions between July 2019 and June 2021. This study was approved by the institutional ethics review committee.

### Inclusion criteria

1. Mammography and ultrasonographically detected palpable breast lesions.
2. Indeterminate breast lesions on mammography and ultrasonography.
3. Extremely dense breast.

### Exclusion criteria

1. Fibrocystic disease of the breast.
2. Fat necrosis, pyogenic abscess, post-traumatic hematoma.
3. Patients with recent breast surgery or biopsy procedures within 12 weeks of duration.
4. Known patient of breast cancer with post-chemo- or radiotherapy.
5. Patient contraindicated to MRI or allergy to IV contrast.

### MRI protocol

Breast MRI scans were done in all 66 female patients using a Philips Ingenia 1.5 Tesla MRI scanner (Philips Medical System, Amsterdam, the Netherlands). MRI scans of breasts were obtained in a prone position with the use of a dedicated breast coil. The various MRI imaging protocol parameters are shown in Table 1.

**Table 1:** Parameters used in various MRI sequences for breast imaging

Sequences	TE (ms)	TR (ms)	Matrix	FOV	Slice thickness (mm)	Flip angle	Others
T1W axial	10–12	500–600	220 × 280	200–220	4	90°	
T2W axial	100–120	3000–4000	220 × 280	200–220	4	90°	
Fat-saturated T2W axial	80–100	3000–4000	220 × 280	200–220	3	90°	
T2W coronal	70–90		220 × 280	200–220	3	90°	
STIR coronal	60–90	2600–4100	220 × 280	200–220	4	90°	TI = 150 ms
DWI axial	55–60	3500–4400		200–220	3	90°	*b* = 800 s/mm2
Dynamic eThrive sequence	8–9	700–800	220 × 152	200–220	3	25°–28°	One pre-contrast and 5 post-contrast series after IV injection of 10 ml gadolinium followed by 10 ml saline
eThrive_HR_Sagittal	3–5	700–800	220 × 340	200–220	3	10°	

*STIR* short tau inversion recovery, *TE* time of echo, *TR* repetition time, *TI* inversion time, *eThrive* enhanced T1 high-resolution isotropic volume excitation, *FOV* field of view

### eThrive dynamic study

The dynamic study consisted of one pre-contrast and five post-contrast series. This complete study was captured within 6–8 min. Each of the pre- and post-contrast series was obtained within 40–45 s. A time gap of about 20–40 s was there between the pre-contrast and post-contrast series and even between the individual post-contrast series. Automatic subtraction images were generated after the subtraction of each of the pre-contrast images from the post-contrast images of each series. The kinetic curves were obtained after placing ROI in the enhancing and non-necrotic portion of the breast lesion.

### Conventional MRI images analysis

Two radiologists reviewed the MRI images. One radiologist had 15 years of experience in MRI and another had 10 years of MRI experience. Both radiologists blindly analyzed the conventional MRI images, DWI, ADC mapping and DCE-MRI parameters. Always DWI and DCE-MRI images were interpreted in conjunction with the conventional MRI images.

The initial morphological analysis of breast lesions was carried out on T1W, T2W and STIR images. The conventional MR images were analyzed for

1. The signal intensity of breast lesion on T1W and T2W images.
2. The largest dimension of breast lesion was measured on T2W and DCE-MRI images.
3. Infiltration into the retromammary space and pectoral muscle was assessed on T2W, STIR, DCE-MRI and DWI images.
4. Axillary lymph nodal enlargement was observed.

### Diffusion-weighted imaging (DWI)

The pattern of diffusion restriction was performed on DWI images with subsequent calculation of ADC values in ADC map image of *b* = 800 s/mm^2^.

### Quantitative DWI and ADC mapping analysis

Two blind-ended radiologists having MRI experience of more than 10 years independently calculated the ADC value in the breast lesions after placing a round or elliptical region of interest (ROI) in the IntelliSpace Portal 9.0 operating system console of Philips. Three uniform sizes ROIs were placed in the ADC map image, and then, the mean ADC value was obtained for analysis.

### Semiquantitative DCE-MRI and kinetic curves

The post-contrast enhancement characteristics of breast lesions were obtained from DCE-MRI and subtraction images with the subsequent creation of kinetic curves in the IntelliSpace Portal 9.0 operating system console of Philips. The maximum relative enhancement (%), time to peak (s), washin rate (l/s) and washout rate (l/s) were calculated from the dynamic contrast-enhanced study (DCE-MRI) after placing the ROI in the enhancing breast lesion. The maximum relative enhancement (%) of the breast lesion with respect to the normal fibroglandular breast tissue (breast lesion to normal parenchymal index) was calculated after placing ROI in the maximum enhancement phase of the DCE-MRI.

DCE-MRI might show three patterns of kinetic curves from an enhancing breast lesion. A progressive increase in contrast uptake throughout the DCE-MRI series showed a type I curve, which strongly suggests benign lesions. Initial contrast uptake in the early post-contrast series of DCE-MRI with plateau phase in the later series showed an indeterminate type II curve, which is suspicious for malignancy [10]. Rapid contrast uptake and rapid contrast washout in DCE-MRI showed a type III curve, which strongly suggests malignancy.

MRI detected various palpable breast lesions that were categorized according to the 5th edition BI-RADS lexicon. Percutaneous core needle biopsy and/or fine needle aspiration cytology was performed in all patients. Breast lesions with more than the BI-RADS 3 category were taken for core needle biopsies. All core needle biopsy samples were obtained within a month of the breast MRI scan. BI-RADS 2 and BI-RADS 3 breast lesions were counted as benign breast lesions while BI-RADS 4 and BI-RADS 5 were counted as malignant breast lesions on MRI and compared with the gold standard histopathological findings.

### Statistical analysis

Data analysis was performed by using SPSS, version 16, Chicago, USA (Statistical Package for Social Science). The mean ADC value of various palpable breast lesions was compared with an independent *t*-test according to the BI-RADS category. Optimal cutoff mean values of each BI-RADS category palpable breast lesions were obtained from the receiver operating characteristic curve (ROC). A *P* value of ≤ 0.05 was considered statistically significant.

## Results

**Fig. 1:**
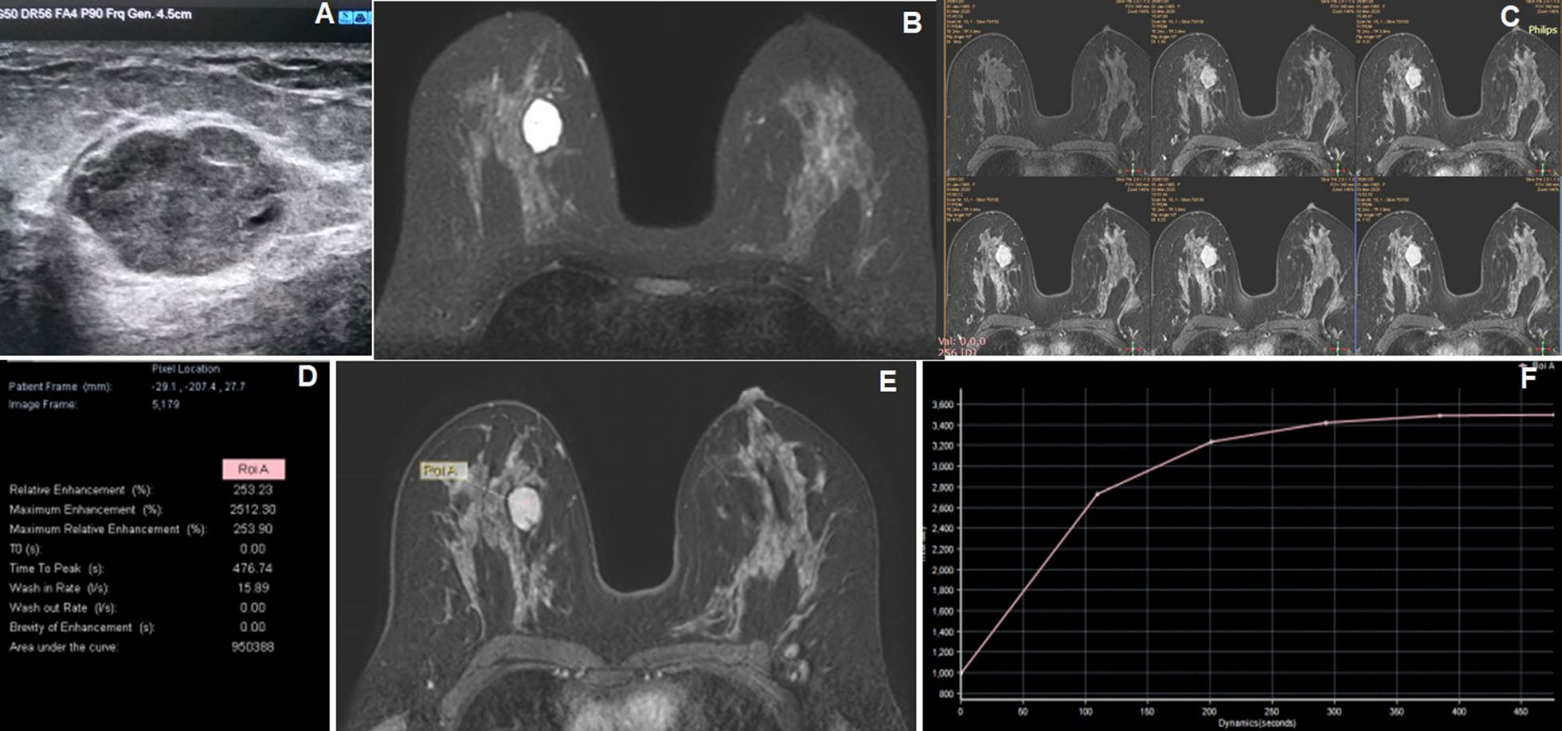
A 35-year-old female presented with a palpable right breast lump. **A** Grayscale USG image reveals a well-defined oval-shaped iso- to hypoechoic lesion. **B** Axial STIR image reveals a mild lobulated hyperintense lesion in the right breast. **C** Axial six-phase dynamic post-contrast MRI (DCE-MRI) reveals progressive homogeneous enhancement of the lesion without necrosis. **D**, **E** and **F** DCE-MRI images show the placement of ROI within the right breast lesion for kinetic curve which shows various values of internal enhancement characteristics of the lesion with a rising type I of kinetic curve. The MRI findings are suggestive of BI-RADS 2 lesion where histopathology showed fibroadenoma

**Fig. 2:**
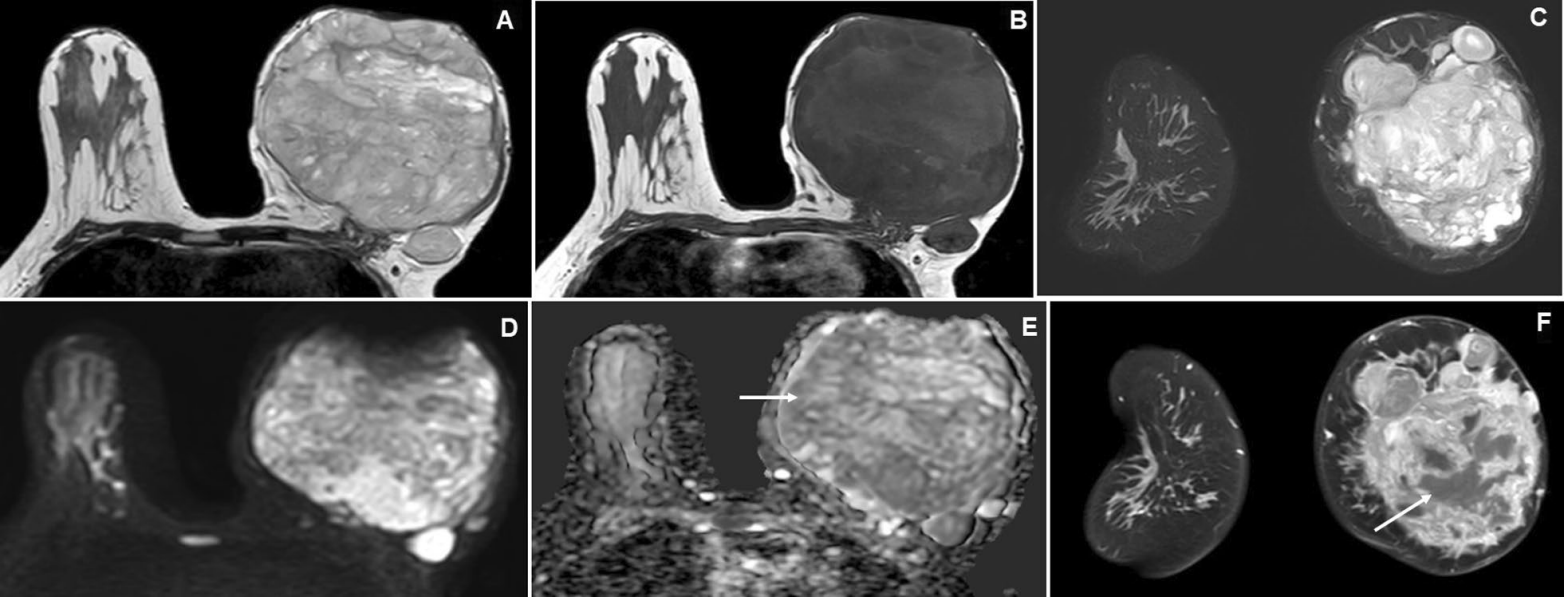
A 22-year-old female presented with a long-standing huge left breast lump. **A**, **B** and **C** Axial T2W, T2W and coronal STIR images reveal a large multi-lobulated T2 hyperintense and T1 iso- to a slight hyperintense lesion in the left breast involving all quadrants. **D** and **E** Axial DWI and ADC map images show variable signal intensities with low ADC value peripherally (arrow). **F** Coronal dynamic post-contrast MRI (DCE-MRI) reveals moderate heterogeneous enhancement of the lesion with variable necrosis (arrow) The MRI findings are suggestive of BI-RADS 4A lesion, where histopathology showed phyllodes tumor

**Fig. 3:**
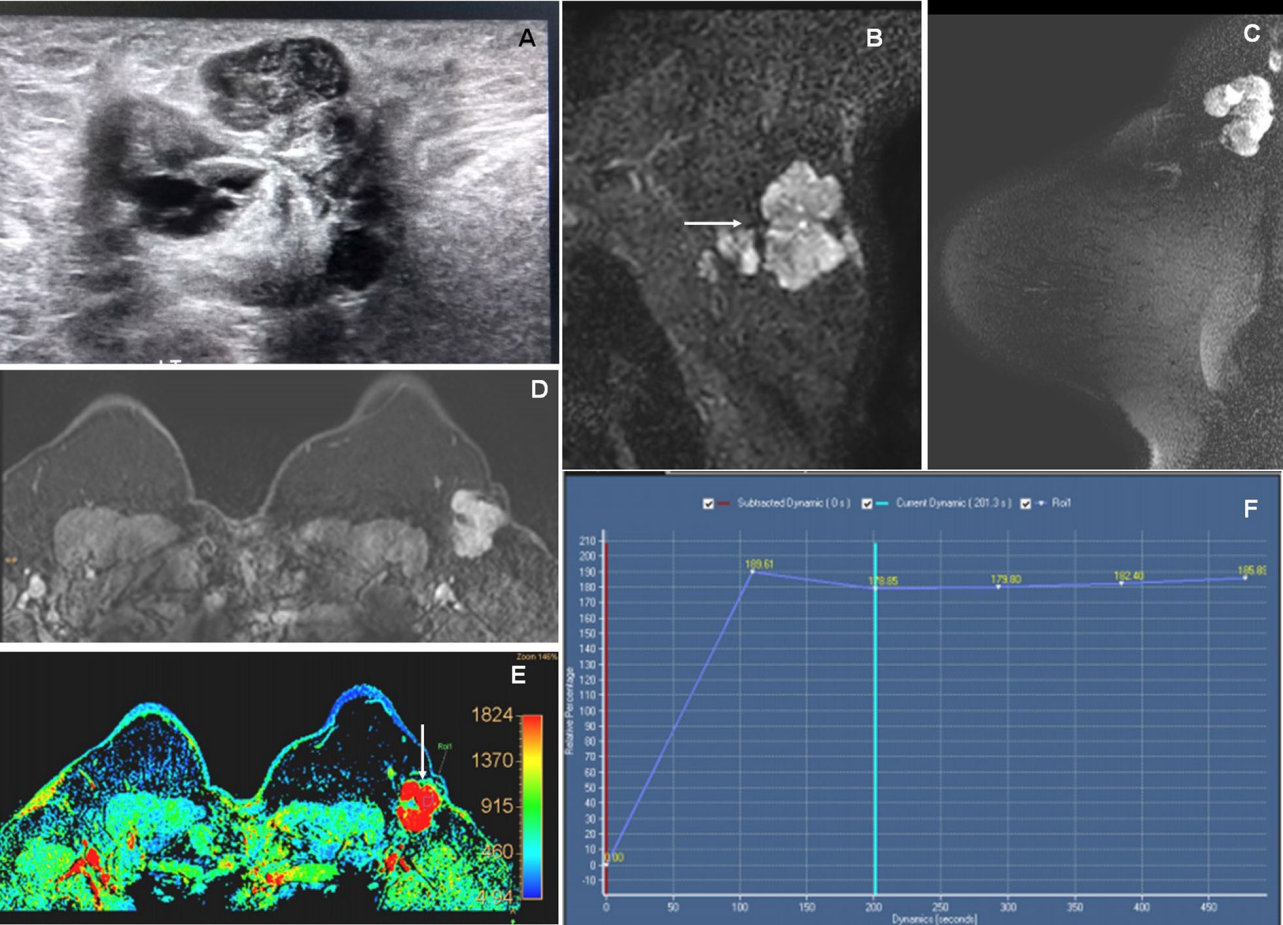
A 35-year-old female presented with a left axillary lump. **A** Grayscale USG image reveals a multi-lobulated mixed heterogeneous echotexture lesion with central hyperechoic and peripheral lobulated isoechoic components in the left axilla. **B** and **C** Coronal and sagittal STIR images reveal multi-lobulated iso- to hyperintense lesions in the left axilla (arrow). **D** and **E** Axial dynamic post-contrast (DCE-MRI) and maximum relative enhancement images reveal homogeneous enhancement of the lesion (arrow). **F** An initial rapid rising with subsequent plateau type of kinetic enhancement curve (type II). The MRI findings are suggestive of BI-RADS 4A lesion, where histopathology showed lobular carcinoma of axillary tail of left breast

**Fig. 4:**
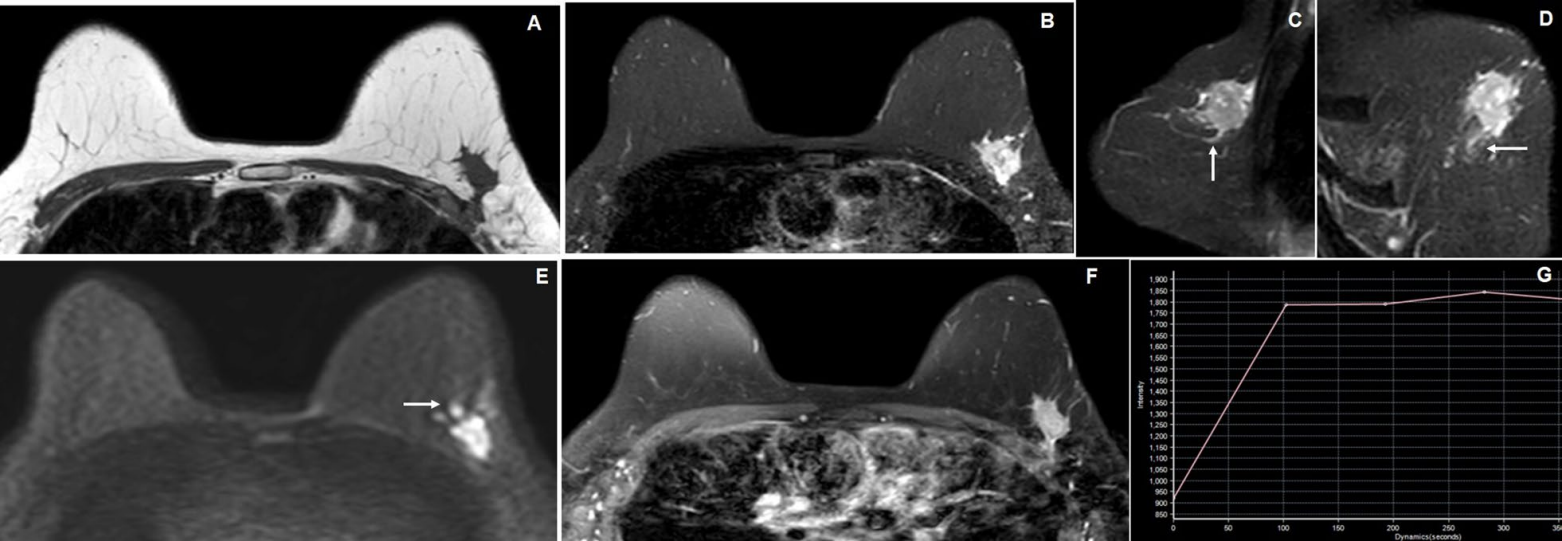
A 56-year-old female with a left breast lump. **A** Axial T1W image reveals an irregular spiculated margined hypointense lesion in the superior lateral quadrant of the left breast. **B**, **C** and **D** Axial, sagittal and coronal STIR images reveal iso- to heterogeneously hyperintense lesion in the left breast with spiculated margins which infiltrates into adjacent fibroglandular breast tissues (arrow). **E** Axial DWI image reveals hyperintense signal intensity of the lesion with irregular nodular hyperintense satellite nodules (arrow). **F** and **G** Axial dynamic post-contrast (DCE-MRI) image reveals homogeneous enhancement of the lesion with enhancing peripheral specules and showing type II kinetic curve. The MRI findings are suggestive of BI-RADS 5 lesion, where histopathology showed invasive ductal carcinoma

In our study sample of 66 females with palpable breast lesions having a mean age of 41.33 ± 13.42[SD] years. Age group distribution showed maximum 22 patients (33.3%) between 31 and 40 years followed by 16 patients (24.2%) in below 30 years, 14 patients (21.2%) in 41–50 years and another 14 patients (21.2%) in above 51 years. According to MRI, BI-RADS 2 category was observed in 28 patients (42.4%) (Fig. 1), BI-RADS 3 in 8 patients (12.1%), BI-RADS 4 in 10 patients (15.2%) (Fig. 2) and BI-RADS 5 in 20 patients (30.3%) (Figs. 3, 4) according to the 5th edition BI-RADS lexicon.

### Histopathological diagnosis

**Fig. 5:**
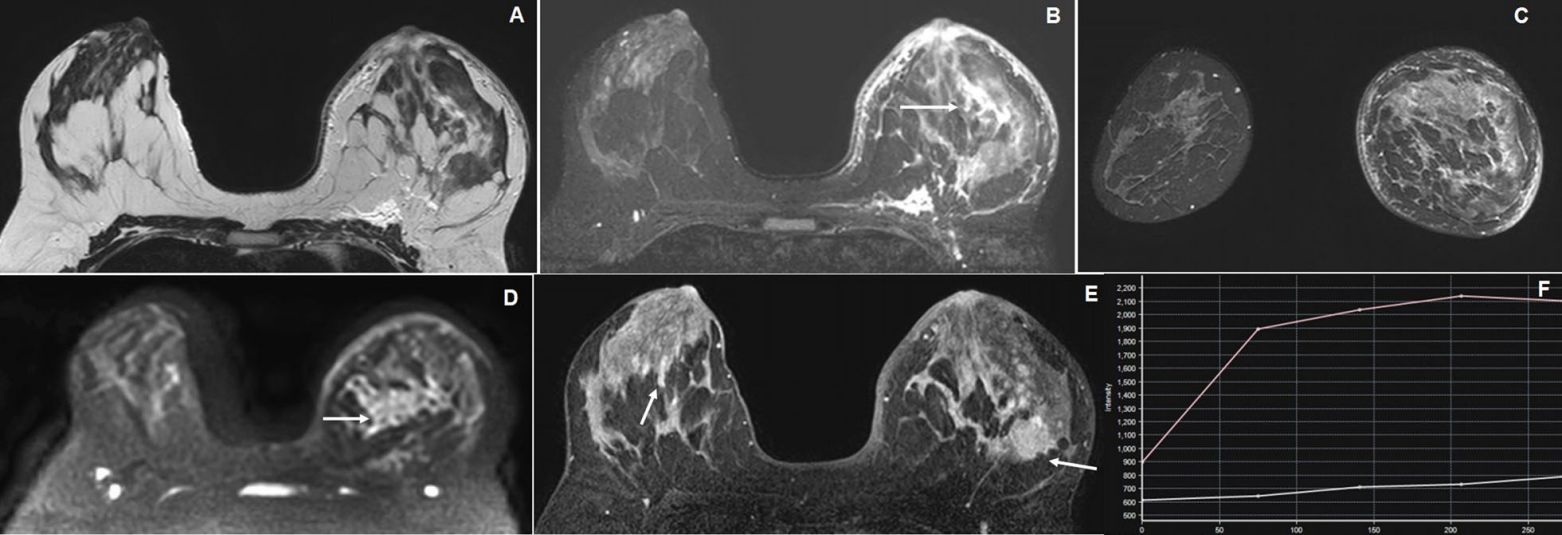
A 45-year-old female presented with a left breast lump. **A**, **B** and **C** Axial T2W, STIR and coronal STIR images reveal increased signal intensities in fibroglandular tissues of left breast, dominantly in superior and inferolateral quadrants (arrow) causing enlargement of left breast with thickening of overlying skin. **D** Axial DWI image shows a heterogeneous diffuse pattern of hyperintensities in the left breast (arrow) with fine nodular hyperintensities in the right breast. **E** and **F** Axial dynamic post-contrast (DCE-MRI) image reveals irregular nodular enhancement of the thickened fibroglandular tissues in both breasts, more on the left side (arrows) where the kinetic curve in the nodular enhanced area shows slow progressive enhancement curve (type I). These lesions were labeled as BI-RADS 2 lesion and were found to be chronic granulomatous mastitis

**Fig. 6:**
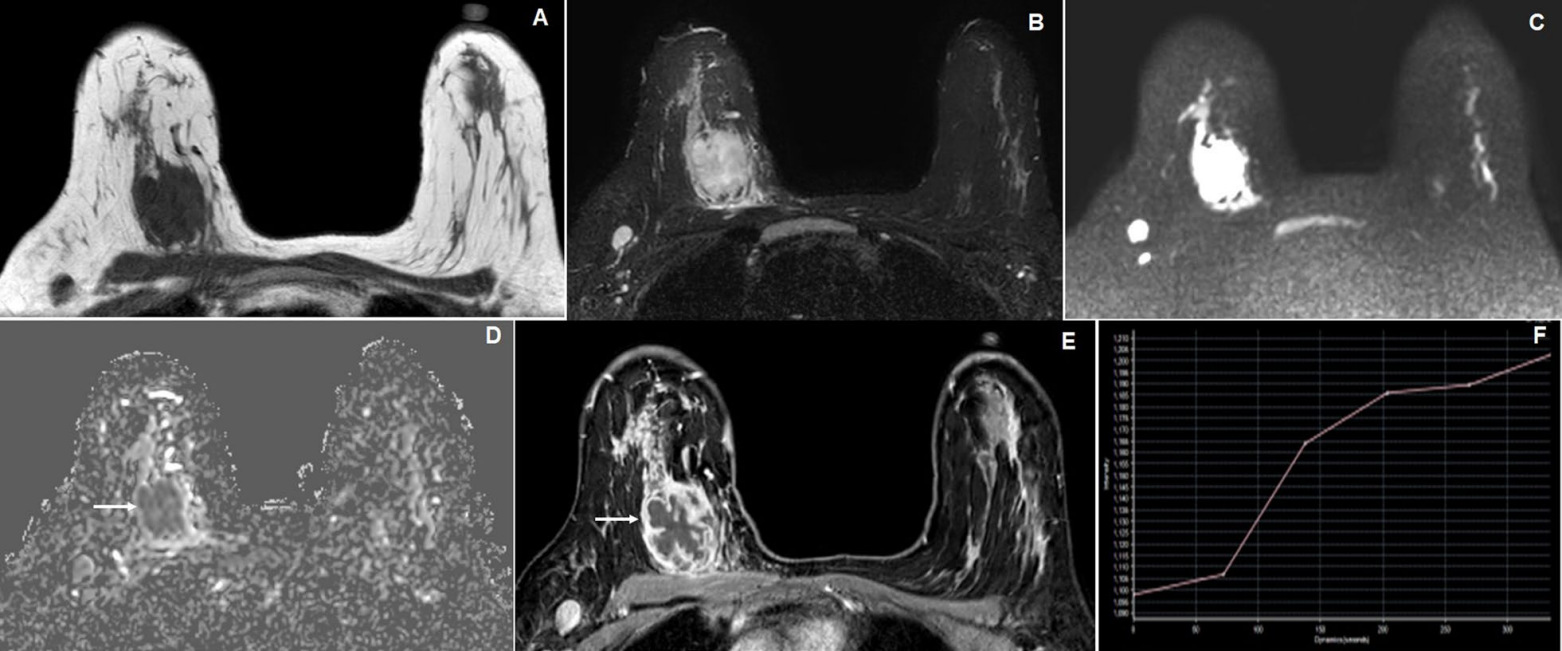
A 32-year-old female presented with a painful right breast lump. **A** and **B** Axial T1W and STIR images reveal T1 hypo and STIR hyperintense lesion in the right breast with edematous adjacent breast fibro glandular tissue. **C** and **D** DWI and exponential ADC images reveal hyperintensities on diffusion-weighted images with low signal intensity on eADC image (arrow). **E** and **F** Axial dynamic post-contrast (DCE-MRI) images reveal thick irregular predominant peripheral enhancement of the lesion with central necrosis and intervening enhancing irregular septa (arrow) and showing type I kinetic curve. The MRI findings are suggestive of BI-RADS 3 lesion, where histopathology showed a Tubercular abscess

Histopathologically, breast carcinomas were found in 28 patients (42.4%), fibroadenomas in 24 patients (36.4%) (Fig. 1), phyllodes in 6 patients (9.1%) (Fig. 2), chronic granulomatous mastitis in 6 patients (9.1%) (Fig. 5) and tubercular abscess in 2 (3%) patients (Fig. 6). All 8 patients of BI-RADS 3 category palpable breast lesions were turned to be benign on histopathology. The mean largest dimension of breast lesion was 47.4 ± 33.1[SD] mm.

### T1 and T2 signal intensity

**Fig. 7:**
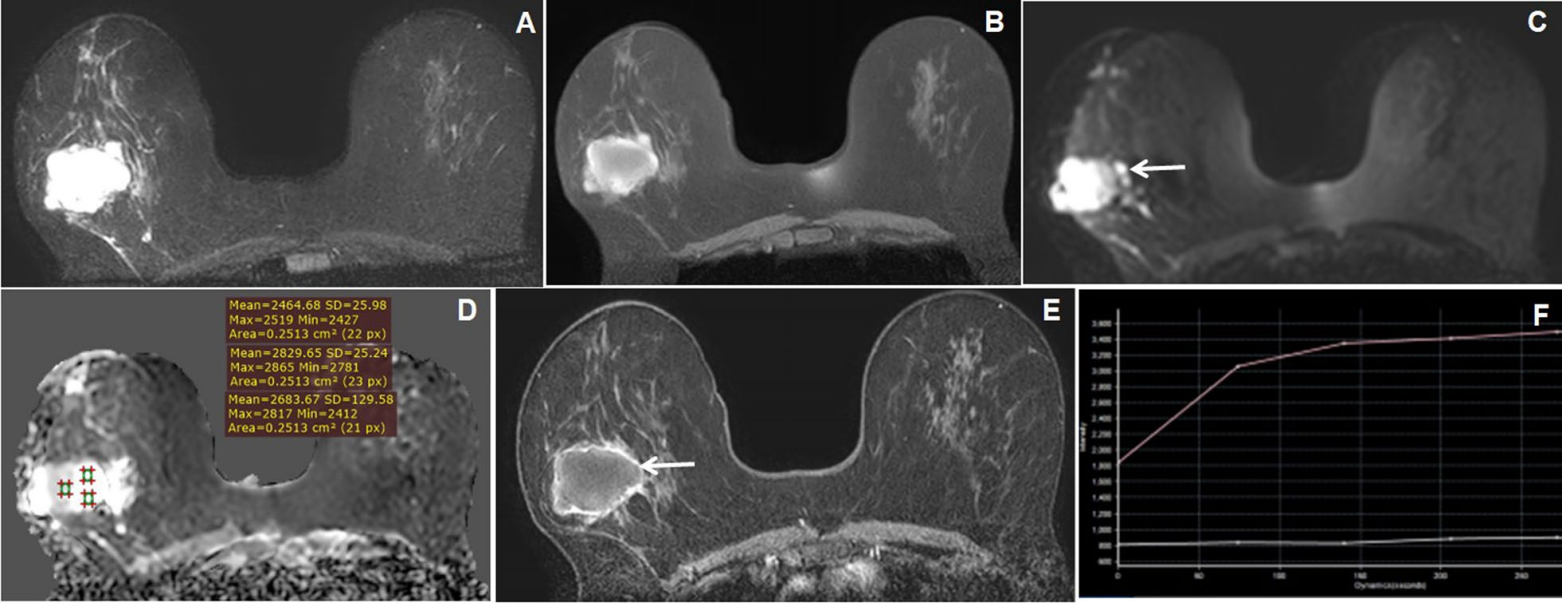
A 48-year-old female presented with a palpable right breast lump. **A** and **B** Axial STIR and fat-suppressed T1W images reveal a lobulated hyperintense lesion in the right breast with peripherally T1 hyperintense and central iso- to slight hypointensity on fat-suppressed T1W image (arrow). **C** and **D** DWI and ADC images reveal hyperintensities on diffusion-weighted images with high signal intensity on ADC image with high ADC values (arrow with ROI placement). **E** and **F** Axial dynamic post-contrast (DCE-MRI) image reveals thick irregular predominant peripheral enhancement of the lesion with central non-enhancing area (arrow) where the kinetic curve in the peripheral enhanced area shows type II curve. The MRI findings are suggestive of BI-RADS 5 lesion, where histopathology showed mucinous carcinoma of the breast

T2WI isointense signal in breast lesions was observed in 16 patients (24.2%) (Fig. 7), T2WI hyperintensities in 32 patients (48.5%) (Fig. 1) and mixed-signal intensities in 18 patients (27.3%) (Fig. 5). On T1W images, isointensities were observed in 34 patients (51.5%), hypointensities in 26 patients (39.4%) and mixed-signal intensities in 6 patients (9.1%) which are shown in Table 2.

**Table 2:** Results of MRI imaging findings in palpable breast lesions in 66 female patients

Parameters	Benign palpable breast lesions (*n* = 36)	Malignant palpable breast lesions (*n* = 30)	*P* value
*T1W appearance*			0.819
Hypointense	16 (24.2%)	10 (15.1%)	
Isointense	18 (27.2%)	16 (24.2%)	
Mixed signals	2 (3%)	4 (6%)	
*T2W appearance*			0.053
Isointense	4 (6%)	12 (18.2%)	
Hyperintense	24 (36.4%)	8 (12.1%)	
Mixed signals	8 (12%)	19 (15.1%)	
*DWI appearance*			0.219
Nodular hyperintense	24 (36.4%)	12 (18.2%)	
Peripheral rim-like hyperintense	0	2 (3%)	
Diffuse heterogeneous hyperintense	12 (18.2%)	16 (24.2%)	
Nodular hypointense	0	0	
*Appearance on post-contrast scan*			0.016
Nodular homogeneous	20 (30.3%)	4 (6%)	
Diffuse heterogeneous	6 (9%)	18 (27.2%)	
Irregular peripheral enhancement with necrosis	10 (15.1%)	8 (12%)	

### Results of qualitative DWI appearance

On diffusion-weighted images, nodular hyperintensity was observed in 24 (36.4%) benign breast lesions and 12 (18.2%) malignant lesions (Fig. 4), peripheral rim-like hyperintensity observed in 2 (3%) malignant breast lesions and diffuse heterogeneous pattern of hyperintensity in 12 (18.2%) of benign breast lesions (Fig. 2) and 16 (24.2%) malignant breast lesions as shown in Table 2. No DWI hypointense lesion was observed.

### Results of quantitative DWI

The mean ADC value of various palpable breast lesions according to the 5th edition BI-RADS lexicon is shown in Table 3. The palpable breast lesions with BI-RADS 2 and BI-RADS 3 were counted as benign lesions on MRI and found in 36 patients (54.5%). Palpable breast lesions of BI-RADS 4 and BI-RADS 5 were counted as malignant breast lesions and found in 30 patients (45.5%). Malignant palpable breast lesions had a mean ADC value of 0.939 ± 0.166[SD] × 10^−3^ mm^2^/s, and benign lesions had 1.891 ± 0.524[SD] × 10^−3^ mm^2^/s, except the mucinous adenocarcinomas showed high ADC value. Unpaired Student *t*-test showed a statistically significant difference between the mean ADC value of malignant and benign breast lesions with a *P* value of 0.0005.

**Table 3:** Mean apparent diffusion coefficient (ADC) values of various palpable breast lesions in 66 female patients

	Parameters	Mean ADC value of breast lesion (× 10^−3^ mm^2^/s)	*P* value
According to American College of Radiology Breast Imaging Reporting and Data System (ACR BI-RADS; 5th edition): 2014	BI-RADS 2	2.056 ± 0.471[SD]	0.0005
	BI-RADS 3	1.314 ± 0.151[SD]	
	BI-RADS 4	0.935 ± 0.119[SD]	
	BI-RADS 5	0.930 ± 0.943[SD]	
	Benign breast lesions	1.891 ± 0.524[SD]	0.0005
	Malignant breast lesions	0.939 ± 0.166[SD]	

**Fig. 8:**
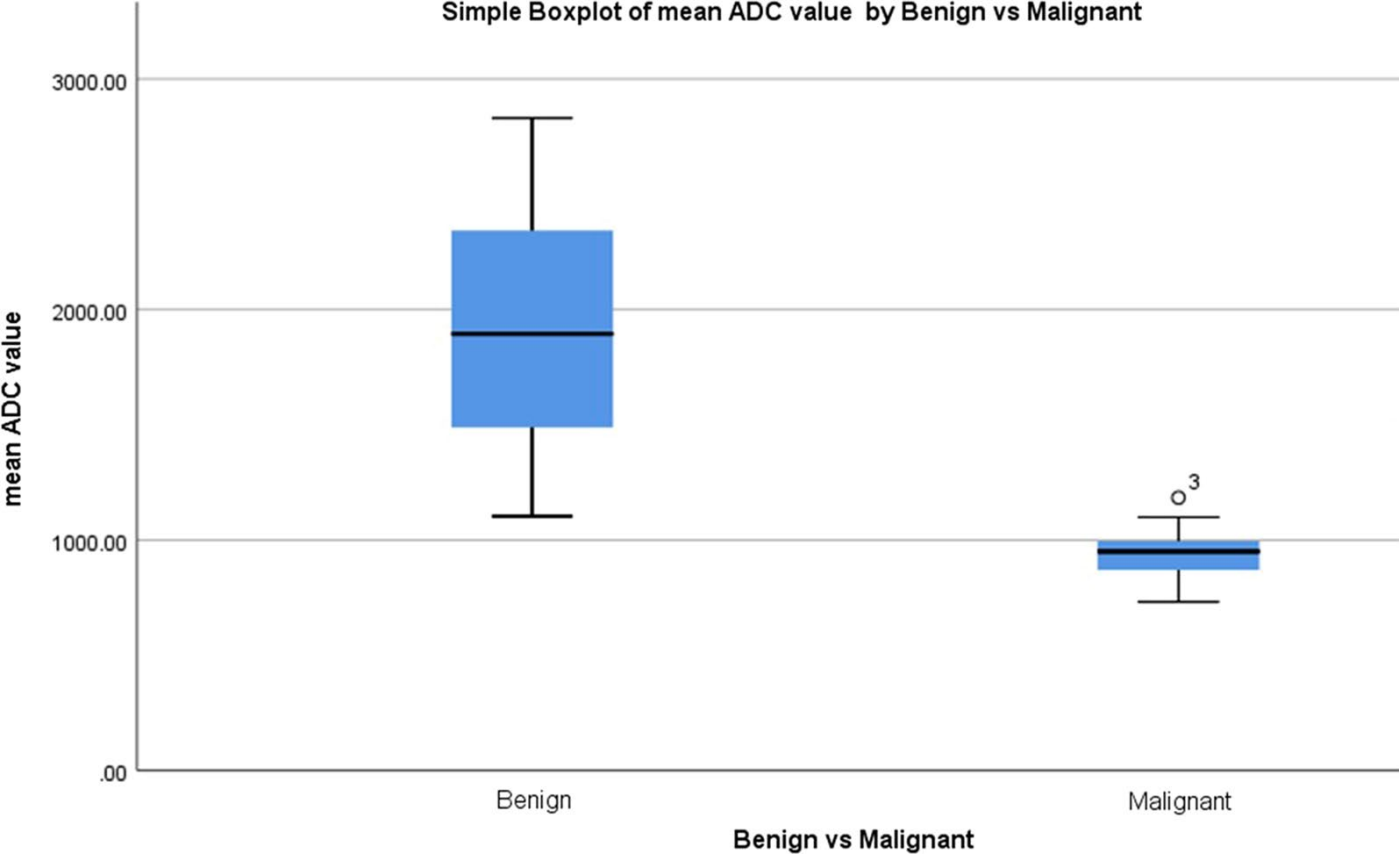
Boxplot showing the distribution of mean ADC value of various benign and malignant palpable breast lesions

The BI-RADS 2 palpable breast lesions had a mean ADC value of 2.056 ± 0.471[SD] × 10^−3^ mm^2^/s, BI-RADS 3 had 1.314 ± 0.151[SD] × 10^−3^ mm^2^/s, BI-RADS 4 had 0.935 ± 0.119[SD] × 10^−3^ mm^2^/s, and BI-RADS 5 had 0.930 ± 0.943[SD] × 10^−3^ mm^2^/s. A statistical significant difference was found between the mean ADC value of various BI-RADS categories of palpable breast lesions with a *P* value of < 0.0005. Boxplot showed the distribution of mean ADC value of various benign and malignant breast lesions (Fig. 8).

### ROC curve analysis of various palpable breast lesions according to the BI-RADS category

**Fig. 9:**
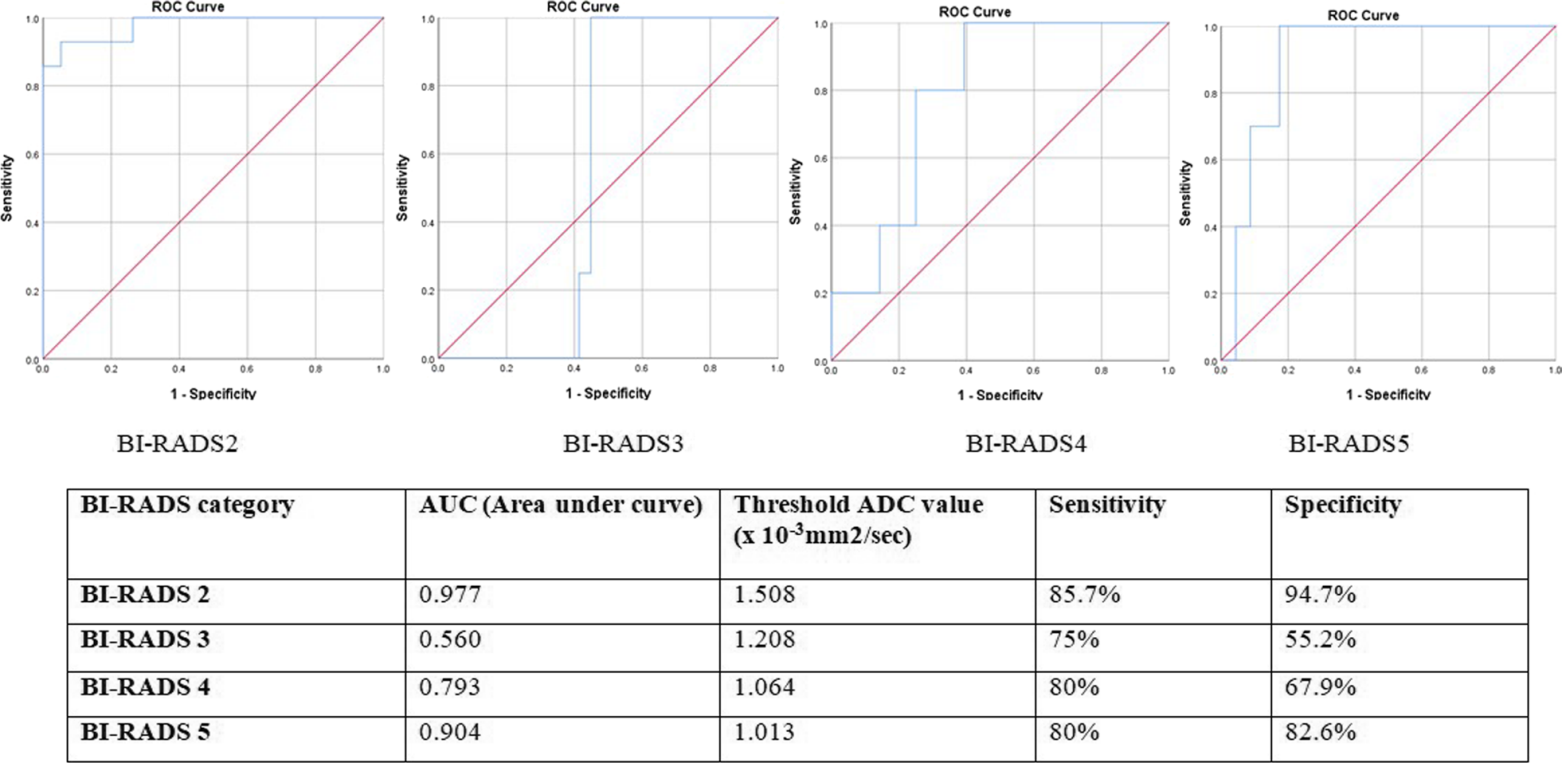
ROC curve analysis showing the cutoff mean ADC value of BI-RADS 2 to BI-RADS 5 category palpable breast lesions

Each BI-RADS category mean ADC values ROC curve analysis is shown in Fig. 9. The BI-RADS 2 category breast lesions showed a cutoff mean ADC value of 1.508 × 10^−3^ mm^2^/s with a sensitivity of 85.7% and specificity of 94.7%, BI-RADS 3 lesions had a cutoff mean ADC value of 1.208 × 10^−3^ mm^2^/s with a sensitivity of 75% and specificity of 55.2%, BI-RADS 4 lesions had cutoff mean ADC value of 1.064 × 10^−3^ mm^2^/s with a sensitivity 80% and specificity of 67.9%, and BI-RADS 5 lesions had cutoff mean ADC value of 1.013 × 10^−3^ mm^2^/s with a sensitivity of 80% and specificity of 82.6%.

### DCE-MRI appearances of breast lesions with internal enhancement characteristics

On DCE-MRI, all breast lesions showed variably increased contrast uptake on all phases after contrast administration. A diffuse nodular pattern of post-contrast enhancement of breast lesions was observed in 20 (30.3%) benign breast lesions and 4 (6%) malignant breast lesions, irregular peripheral post-contrast enhancement was observed in 10 (15.2%) benign breast lesions and 8 (12%) malignant breast lesions, and diffuse heterogeneous post-contrast enhancement pattern was observed in 6 (9%) benign breast lesions and 18 (27.3%) malignant breast lesions, which is shown in Table 2.

The maximum relative enhancement (%) of the breast lesion with respect to the normal fibroglandular breast tissue (breast lesion to normal parenchymal index), time to peak (s), washin rate (l/s), washout rate (l/s) were calculated from DCE-MRI according to the BI-RADS category and are shown in Table 4. Table 5 shows the comparison between the BI-RADS MRI category and pathological findings of various palpable breast lesions.

**Table 4:** Results of dynamic contrast-enhanced (DCE-MRI) MR imaging findings in 66 patients of palpable breast lesions

Parameters	Benign breast lesions (*n* = 36)	Malignant Breast lesions (*n* = 30)	*P* value
Largest dimension of lesion (mm)	42.19 ± 29.04[SD]	53.67 ± 37.43[SD]	0.329
Maximum relative enhancement (%)	77.06 ± 68.5[SD]	115.6 ± 50.1[SD]	0.080
Time to peak (s)	312.8 ± 121.1[SD]	296.9 ± 125.1[SD]	0.715
Washin rate (l/s)	8.17 ± 7.8[SD]	8.6 ± 5.9[SD]	0.868
Washout rate (l/s)	0.46 ± 0.65[SD]	1.89 ± 0.51[SD]	0.0005

**Table 5:** Comparison between the MRI findings and histopathological findings in terms of BI-RADS staging of palpable breast lesions

On combined DCE-MRI + DWI MRI according to the ACR BI-RADS; 5th edition: 2014 (*n* = 66)	On histopathological examination (HPE); (*n* = 66)	HPE diagnosis
BI-RADS 2 and BI-RADS 3 diseases (*n* = 36)	Benign lesions (*n* = 38)	Fibroadenoma (*n* = 24) Phyllodes (*n* = 6) Chronic granulomatous mastitis (*n* = 6) Tubercular mastitis (*n* = 2)
BI-RADS 4 and BI-RADS 5 diseases (*n* = 30)	Malignant lesions (*n* = 28)	Carcinoma (*n* = 28)

### Kinetic curves on DCE-MRI

Type I kinetic curve was observed in 34/36 patients (94.4%) with benign breast lesions (Fig. 1) and 2/30 patients (6.6%) with malignant breast lesions. Type II kinetic curve was observed in 2/36 patients (5.5%) with benign breast lesions and 24/30 patients (80%) with malignant breast lesions. Type III kinetic curve was observed in 4/30 patients (13.3%) with malignant breast lesions.

## Discussion

Mammography is the imaging tool for breast cancer screening for early detection of breast cancer. MRI cannot be used as a screening imaging modality for breast cancer because of the cost and readily non-availability of the MRI. So, an MRI of breasts indicated in unequivocal breast lesions, which are detected as indeterminate on mammography and or ultrasonography.

The conventional breast MRI with DCE-MRI provides the morphological and enhancement kinetics of the breast lesions and provides information regarding tumor physics, vascularity and vascular permeability with high sensitivity and moderate specificity [11]. In our study, we evaluated the use of DWI with ADC mapping along with conventional and DCE-MRI to increase the diagnostic efficacy in the differentiation of various benign and malignant breast lesions. DWI is affected by tumor cellularity, presence of edema, fibrosis and tumor necrosis, while ADC mapping quantifies the diffusion characteristics of the tumor [12], where malignant tumors showed low ADC values, except mucinous tumors showed high ADC value [13].

Enhancement pattern on dynamic contrast-enhanced MRI of a palpable focal breast mass lesion is the most efficient feature to differentiate benign from malignant breast lesions [14–16]. In our study sample, 18 (27.2%) palpable malignant breast lesions showed heterogeneous post-contrast enhancement on DCE-MRI. The degree of vascularity of a breast lesion on DCE-MRI can be attained from the kinetic time–intensity curve.

Previous studies showed increased sensitivity of kinetic curves for differentiation of benign from malignant breast lesions [17]. Roganovic et al. [17] found type III kinetic curves in 86% of malignant breast lesions. In our study sample, type I kinetic curves were seen in 94.4% of benign lesions, type II in 5.5% of benign and 80% of malignant lesions, and type III in 13.3% of malignant palpable breast lesions.

Perfusion effect on DWI imaging seen when b-value below 400 s/mm^2^ was used [18]. The malignant lesions had higher perfusion than benign lesions. So, higher b-value DWI imaging is more useful for differentiating benign from malignant lesions. In our study, all palpable breast lesions showed a variable pattern of signal intensities. Nodular hyperintensity was observed in 66.7% of benign and 40% of malignant palpable breast lesions while diffuse heterogeneous hyperintensity in 53.3% of malignant and 33.3% of benign breast lesions. The results are almost similar to a study conducted by Youssef et al. [6].

Recent studies concluded that DWI with ADC mapping had a promising role in the differentiation of palpable benign and malignant breast lesions and these studies revealed significantly lower ADC values in malignant lesions than in benign lesions [11, 13, 19–26]. In our study sample, the mean ADC value of palpable malignant breast lesion was 0.939 ± 0.166[SD] × 10^−3^ mm^2^/s and benign lesion was 1.891 ± 0.524[SD] × 10^−3^ mm^2^/s. The mean ADC value in BI-RADS 2 category lesion was 2.056 ± 0.471[SD] × 10^−3^ mm^2^/s, BI-RADS 3 was 1.314 ± 0.151[SD] × 10^−3^ mm^2^/s, BI-RADS 4 was 0.935 ± 0.119[SD] × 10^−3^ mm^2^/s, and BI-RADS 5 was 0.930 ± 0.943[SD] × 10^−3^ mm^2^/s.

In our study sample, all 28 patients of BI-RADS 2 and 8 patients BI-RADS 3 categories were turned to be benign in nature on histopathological examination, while 28 out of 30 patients of BI-RADS 4 and BI-RADS 5 turned to be malignant on histopathological examination.

In our study sample, the histopathologically 28 patients (42.4%) with breast carcinomas showed low ADC value, except for patients with mucinous carcinoma of the breast showed a higher ADC value than the other types of breast carcinomas. This result was consistent with previous study results [27].

In our study sample, the combined use of conventional MRI, DWI and DCE-MRI had a sensitivity of 93.94% and specificity of 93.94% as compared to the histopathological findings shown in Table 5. These findings are well correlated with the previous study of Kul et al. [8]. El Bakry et al. [28] found DCE-MRI alone had a sensitivity of 91.7% and specificity of 84.2% and DWI alone had a sensitivity of 94.4% and specificity of 92.1% for differentiating various breast tumors.

### Limitation of the study

The study sample is relatively small, therefore, a larger study sample size is necessary to confirm these findings in the future. Diffusion-weighted imaging is more prone to susceptibility artifacts and image distortion, therefore causing difficulty in the detailed characterization of the small breast lesions.

The Global COVID-19 pandemic impacted our study, because of less number of patients undergoing breast imaging, delays in breast imaging, delay in undergoing breast biopsies with indeterminate palpable breast lesions and lost few patients posted for breast biopsies.

## Conclusions

Diffusion restriction on diffusion-weighted images and low ADC value in ADC mapping of a breast tumor favors more toward malignant nature except for mucinous tumors along with type II and or III kinetic curves on DCE-MRI. So, the combined use of ADC mapping and DCE-MRI imaging increases the accuracy of conventional MRI imaging in the characterization and differentiation of various palpable breast lesions compared with DCE-MRI or DWI alone.

## Data Availability

The datasets used and analyzed during the study are available from the corresponding author on reasonable request.

## Abbreviations

MRI: Magnetic resonance imaging
DCE-MRI: Dynamic contrast-enhanced MRI
STIR: Short tau inversion recovery
DWI: Diffusion-weighted imaging
eThrive: Enhanced T1 high-resolution isotropic volume excitation
